# Identification and clinical validation of NUSAP1 as a novel prognostic biomarker in ovarian cancer

**DOI:** 10.1186/s12885-022-09753-4

**Published:** 2022-06-23

**Authors:** Rui Gou, Mingjun Zheng, Yuexin Hu, Lingling Gao, Shuang Wang, Ouxuan Liu, Xiao Li, Liancheng Zhu, Juanjuan Liu, Bei Lin

**Affiliations:** 1 Department of Obstetrics and Gynecology, Shengjing Hospital of China Medical University, Liaoning, 110004 China; 2 Key Laboratory of Maternal-Fetal Medicine of Liaoning Province, Key Laboratory of Obstetrics and Gynecology of Higher Education of Liaoning Province, Liaoning, China; 3 Department of Obstetrics and Gynecology, University Hospital, LMU Munich, Marchioninistr 15, 81377 Munich, Germany

**Keywords:** NUSAP1, Ovarian cancer, Prognostic biomarker, Immunohistochemistry, Immune infiltration

## Abstract

**Background:**

Nucleolar and spindle-associated protein 1 (NUSAP1) was shown to be involved in cell cycle regulation in cancer. However, its prognostic value and underlying mechanism in ovarian cancer remain unclear.

**Methods:**

Oncomine, TCGA, CCLE, and UALCAN databases were used to analyze the expression level of NUSAP1 in ovarian cancer. The Kaplan–Meier plotter database was used to evaluate its prognostic value. The results from these analyses were further validated using immunohistochemical assay. The potential molecular mechanism of *NUSAP1* in ovarian cancer was assessed with respect to homologous recombination repair, mismatch repair, and immunology using different databases.

**Results:**

Database analyses and experimental results demonstrated that NUSAP1 was highly expressed in ovarian cancer, its levels being correlated with the FIGO stage. High NUSAP1 expression was an independent risk factor affecting the prognosis of patients with epithelial ovarian cancer. Moreover, *NUSAP1* was associated with cell cycle, DNA replication, homologous recombination, and p53 signaling pathway. A positive correlation was identified between the expression of *NUSAP1* and *BRCA1/2* in ovarian cancer. In addition, *NUSAP1* was associated with the expression of DNA mismatch repair genes and immune cell infiltration.

**Conclusions:**

NUSAP1 may be a valuable prognostic marker, as well as a novel biomarker for evaluating the response to immunotherapy of patients with ovarian cancer.

## Background

Ovarian cancer is a common malignant tumor of the female reproductive system. The Global Cancer Observatory 2020 statistics reported that ovarian cancer causes approximately 313,959 new cases and 207,252 deaths worldwide each year [1]. Surgery and platinum-based chemotherapy are routine therapy options for ovarian cancer [2, 3]. Although the prognosis of early-stage ovarian cancer is favorable with treatment, nearly 75% of women present with International Federation of Gynecology and Obstetrics (FIGO) stage III–IV, which is associated to reduced 5-year survival rates [4]. Therefore, it is important to clarify the mechanism of ovarian tumorigenesis and explore new molecular markers.

The nucleolar and spindle-associated protein 1 (NUSAP1) is a kind of microtubule- and chromatin-binding protein involved in the regulation of spindle formation and stability, chromosome segregation, and cytokinesis [5, 6]. Its expression peaks when cells transition from the G2 phase of the cell cycle to the mitotic phase [7]. NUSAP1 is highly expressed in multiple malignant tumors, such as breast cancer [8], hepatocellular carcinoma [9], esophageal squamous cell carcinoma [10], and glioblastoma [11]. High NUSAP1 expression promotes malignant biological behaviors, such as tumor invasion and metastasis, and is associated with poor prognosis in patients with astrocytoma [12], breast cancer [13], colon cancer [14], and cervical cancer [15]. In addition, NUSAP1 maintains resistance to the toxic effects of anti-tubulin chemotherapeutics [16]. Therefore, NUSAP1 has extensive research prospects in cancer. However, its prognostic value and underlying specific molecular involvement in ovarian cancer have not yet been clarified.

Our study aimed to explore the clinical significance of NUSAP1 and provide a new research direction for finding biomarkers of ovarian cancer. This study is the first to report the prognostic value of NUSAP1, as well as its involvement in signaling pathways and correlation with immune cell infiltration in ovarian cancer.

## Methods

### Data extraction from Oncomine, TCGA and CCLE databases

The Oncomine database (http://www.oncomine.org) was used to validate the *NUSAP1* expression in cancer [17]. The criteria were set as previously reported [18]. Furthermore, we explored the expression of *NUSAP1* in multiple cancers from The Cancer Genome Atlas (TCGA) program (https://www.cancer.gov/tcga).

The Cancer Cell Line Encyclopedia (CCLE) project (https://www.broadinstitute.org/ccle) includes 1457 cell lines and 84,434 genes, and provides information on the genetic and pharmacologic characterization of cancer models [19]. It was used to validate the expression profiles of *NUSAP1* in multiple cancer cell lines, and the correlation between the expression of *NUSAP1* and *BRCA1/2*.

### Data extraction from UALCAN and HPA databases

UALCAN (http://ualcan.path.uab.edu) uses data from Clinical Proteomic Tumor Analysis Consortium (CPTAC) to analyze protein expression [20]. CPTAC integrates the data of genomic and proteomic to improve the understanding of the molecular basis of cancer. The database was used to validate the protein levels of NUSAP1 in ovarian cancer.

The Human Protein Atlas (HPA) database (https://www.proteinatlas.org) provides tissue and cell distribution information of proteins [21]. The protein expression of NUSAP1 in ovarian cancer was validated using immunohistochemistry assays.

### Kaplan–Meier plotter analysis

The Kaplan–Meier plotter (https://kmplot.com/analysis) can be used to assess the prognosis information of 54,675 genes in different cancer types [22]. In this study, the Kaplan–Meier plotter was used to analyze the prognostic value of *NUSAP1* by calculating its respective hazard ratios (HRs) and 95% confidence intervals (CIs). Log-rank *P* < 0.05 indicated statistically significant differences.

### Sample source and clinical data

In this study, 114 ovarian tissues obtained between 2008 and 2012 from the Shengjing Hospital of China Medical University were embedded in paraffin. Patients included in the study did not receive radiation therapy, chemotherapy, or hormone therapy before surgery. Comprehensive staging operation and cytoreductive surgeries for ovarian cancer were performed in early and advanced stage cases, respectively. Complete clinicopathological data was available. All pathological sections were re-examined and clearly diagnosed by pathologists using the diagnostic criteria stated in the World Health Organization 2014 classification before follow-up. Cystadenomas or cystadenofibromas with > 10% borderline histology were classified as borderline ovarian tumor. The 114 samples included 78 epithelial ovarian cancer cases, 14 epithelial ovarian borderline tumors, 10 epithelial ovarian benign tumors, and 12 normal ovarian tissues. No significant difference in age among the four groups was observed (*P* > 0.05): the malignant tumor group had a median age of 52 years (range: 27–83 years), for the borderline tumor group was of 43 years (19–84), for the benign tumor group was of 48 years (28–61), and for the normal ovarian group was of 43 years (32–65). Within the malignant tumor group, 23 cases were grade 1, 20 cases were grade 2, and 35 cases were grade 3. According to the 2009 FIGO standards, 34 patients had stage I-II and 44 had stage III-IV ovarian cancer. Evaluation of lymph node metastases revealed metastases in 18 cases, no metastasis in 33 cases, and no lymphadenectomy in 27 cases. Within the borderline tumor group, there were seven samples of serous borderline tumor and seven samples of mucinous borderline tumor. Within the benign tumor group, there was one sample of benign serous tumor and nine samples of benign mucinous tumor.

### Immunohistochemistry (IHC) assay

IHC assay and scoring method were carried out according to previously published methods [18, 23]. The samples were dewaxed with xylene and rehydrated with sequential solutions of decreasing ethanol concentrations. Thereafter, the immunostaining permeation solution was used to permeate the membrane. After inhibiting the endogenous peroxidase activity, antigen retrieval was performed with heat treatment. After cooling, the samples were blocked with goat serum for 30 min at 37 °C. A working concentration of 1:100 polyclonal antibody against NUSAP1 (12024–1-AP; Proteintech, Wuhan, China) was used to evaluate NUSAP1 expression in ovarian cancer. After overnight incubation at 4 °C, biotinylated secondary antibodies were added for 30 min at 37 °C. 3,3′-Diaminobenzidine was used to visualize the antibody complexes. The sections were then stained with hematoxylin, dehydrated, and examined. Positive control and negative control were set for each batch. All sections were visualized under a Nikon Eclipse Ci microscope (Tokyo, Japan) and captured by NIS-Elements F software. The final scores resulted from multiplying the staining and percentage of stained cells scores: 0–2 scores (–), 3–4 scores ( +), 5–8 scores (+ +), and 9–12 scores (+ + +). Two senior pathologists scored each tissue slice independently to reduce errors.

### Gene set enrichment analysis (GSEA)

According to the median expression of *NUSAP1*, the TCGA-OV data set was divided into high and low expression groups. GSEA version 3.0 was used to analyze the data [24]. We downloaded the C2.cp.kegg.v6.1.symbols.gmt data cluster from the Molecular Signatures Database, and performed enrichment analysis. The number of random combinations was set to 1,000.

### Metascape database analysis

Metascape (http://metascape.org) is an online platform that provides gene enrichment, biological process annotation, and protein interaction network information [25]. Metascape was used to evaluate the functional and pathway enrichment of *NUSAP1* and its co-expressed genes.

### cBioPortal database analysis

The cBioPortal database (https://www.cbioportal.org) is an online platform that provides information on DNA copy numbers, DNA methylation, mRNA and microRNA expression, and nonsynonymous mutations [26]. The database was used to analyze the correlation between *NUSAP1* and *BRCA1/2* in ovarian cancer.

### Immune infiltration analysis

The TIMER database (http://timer.cistrome.org) contains the information of immune cell infiltration in different cancer types [27]. We evaluated the correlation of *NUSAP1* expression with immune infiltrates, and the effect of *NUSAP1* copy number variations on the infiltration of immune cells. Furthermore, the TISIDB database (http://cis.hku.hk/TISIDB), which integrates multiple public databases to provide the information about tumor-immune system interactions, was employed to explore the distribution of *NUSAP1* expression across molecular subtypes and immune subtypes in ovarian cancer [28].

### Statistical analysis

SPSS 22.0 software (IBM Corporation, Armonk, NY, USA) was used to analyze the data. Chi-squared and Fisher’s exact probability tests were performed to analyze the count data, and Student’s *t*-test was used to analyze the measurement data. Survival curves were analyzed via Kaplan–Meier and log-rank tests. The Cox regression model was used to analyze the relationships between NUSAP1 expression and clinicopathological parameters. *P* < 0.05 was the cutoff value established for statistical significance.

## Results

### Validation of NUSAP1 expression level in different databases

**Fig. 1:**
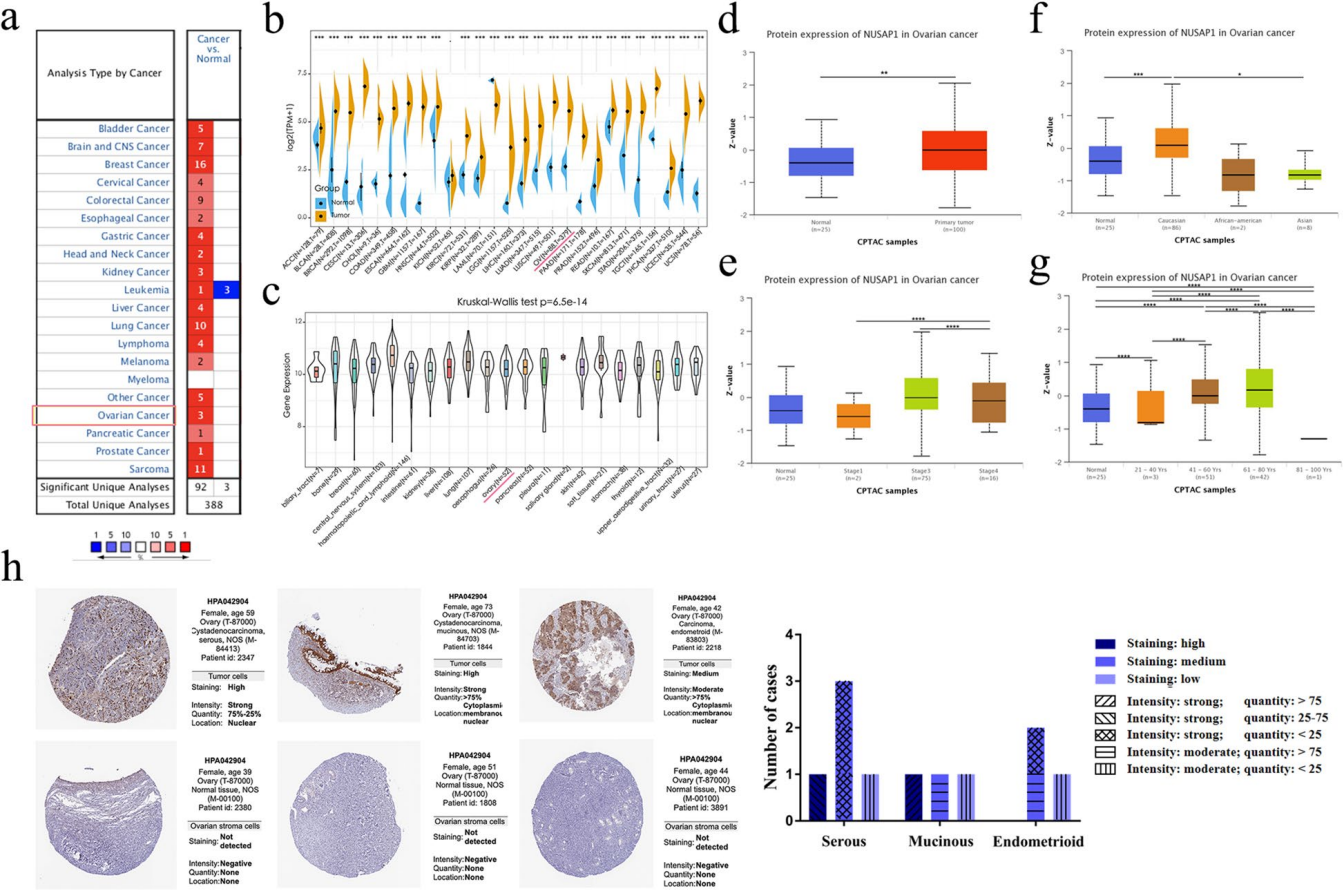
NUSAP1 expression in multiple databases. **a** The expression level of *NUSAP1* in various types of cancer based on Oncomine data. The color intensity of red or blue is directly proportional to the significance level of upregulation or downregulation. The cell number represents the number of datasets that meets the screening criteria. **b** The mRNA expression levels of *NUSAP1* in various cancer types and corresponding normal tissues based on TCGA data. **c** The mRNA expression levels of *NUSAP1* in multiple cancer cell lines based on CCLE database. **d-g** The protein expression level of NUSAP1 based on UALCAN database. **h** Representative photomicrographs and bar graphs of NUSAP1 expression base on HPA data. *P < 0.05; **P < 0.01; ***P < 0.001; ****P < 0.0001

We initially evaluated *NUSAP1* expression in multiple cancers through the Oncomine database (Fig. 1a). Of these studies, 92 showed that the expression of *NUSAP1* was significantly upregulated, including three studies in ovarian cancer. The difference in expression between multiple cancer and normal tissues was evaluated through the TCGA database (Fig. 1b). Ovarian cancer datasets showed that *NUSAP1* expression was significantly upregulated in ovarian cancer. The data of each tumor cell line were downloaded from the CCLE database and similar results were obtained (Fig. 1c). Furthermore, the UALCAN database was used to analyze the expression of NUSAP1 in ovarian cancer and its correlation with clinical parameters. NUSAP1 expression was significantly increased in ovarian cancer compared with that of normal tissues, especially in FIGO stage III (Fig. 1d, e). In addition, the level of NUSAP1 was higher in Caucasian people and patients aged between 61 and 80 years old compared with other subgroups (Fig. 1f, g). The HPA database evaluated the level of NUSAP1 in ovarian serous carcinoma, mucinous carcinoma, endometrioid carcinoma, and normal tissues using immunohistochemical data. There were different degrees of staining in ovarian cancer tissues, but no staining in normal tissues. Representative photomicrographs of various tissue samples were selected for protein expression validation (Fig. 1h).

### Relationship between NUSAP1 expression level and ovarian cancer prognosis

**Fig. 2:**
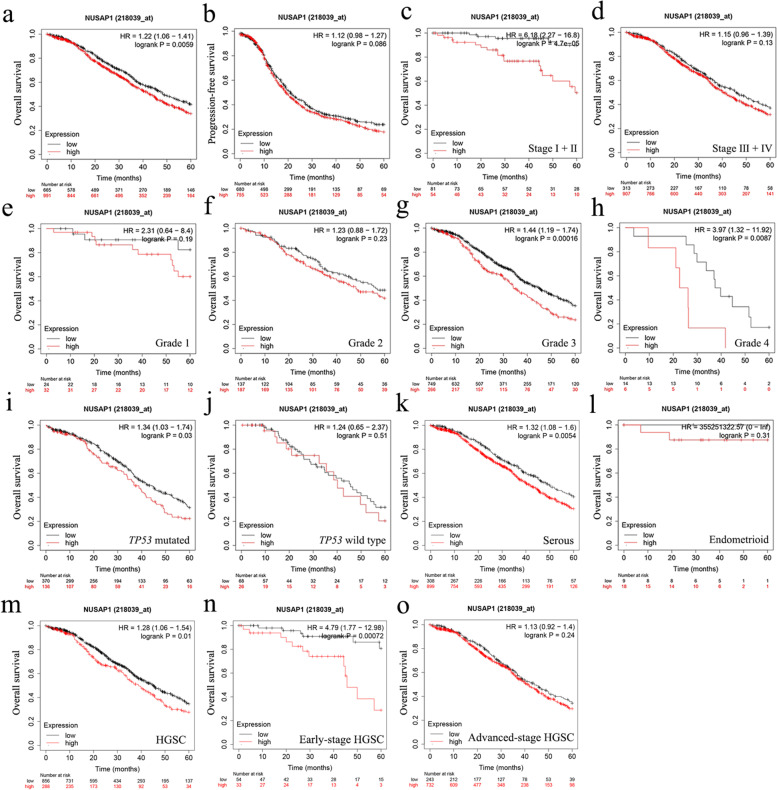
Relationship between *NUSAP1* expression and prognosis of patients with ovarian cancer. **a** Relationship between *NUSAP1* expression and OS in patients with ovarian cancer. **b** Relationship between *NUSAP1* expression and PFS in patients with ovarian cancer. **c–d** Prognostic significance of *NUSAP1* in ovarian cancer with different FIGO stages. **e–h** Prognostic significance of *NUSAP1* in ovarian cancer with different grades. **i–j** Prognostic significance of *NUSAP1* in ovarian cancers harboring or not *TP53* mutations. **k–l** Prognostic significance of *NUSAP1* in ovarian cancer with different pathological subtypes. **m–o** Prognostic significance of *NUSAP1* in high-grade serous ovarian cancer. *P*-values were calculated using the log-rank test

The correlation between *NUSAP1* expression and patient prognosis was analyzed using the Kaplan–Meier plotter database. Results showed that high *NUSAP1* expression was correlated with poor overall survival (OS) (Fig. 2a). However, the analysis of progression-free survival (PFS) did not yield similar results (Fig. 2b). *NUSAP1* upregulation indicated poor OS in patients with FIGO stage I+II (Fig. 2c, d). In addition, *NUSAP1* upregulation was correlated with poor OS in patients with grades 3 and 4 (Fig. 2e, f, g, h). A significant correlation was observed between upregulation of *NUSAP1* and poor OS in patients harboring *TP53* mutations (Fig. 2i, j). Upregulation of *NUSAP1* was correlated with poor OS in ovarian serous cancer, but not in ovarian endometrioid cancer (Fig. 2k, l). Further analysis showed that high *NUSAP1* expression conferred poor OS in high-grade serous ovarian cancer (HGSC) (Fig. 2m), especially in the early-stage (Fig. 2n, o).

**Fig. 3:**
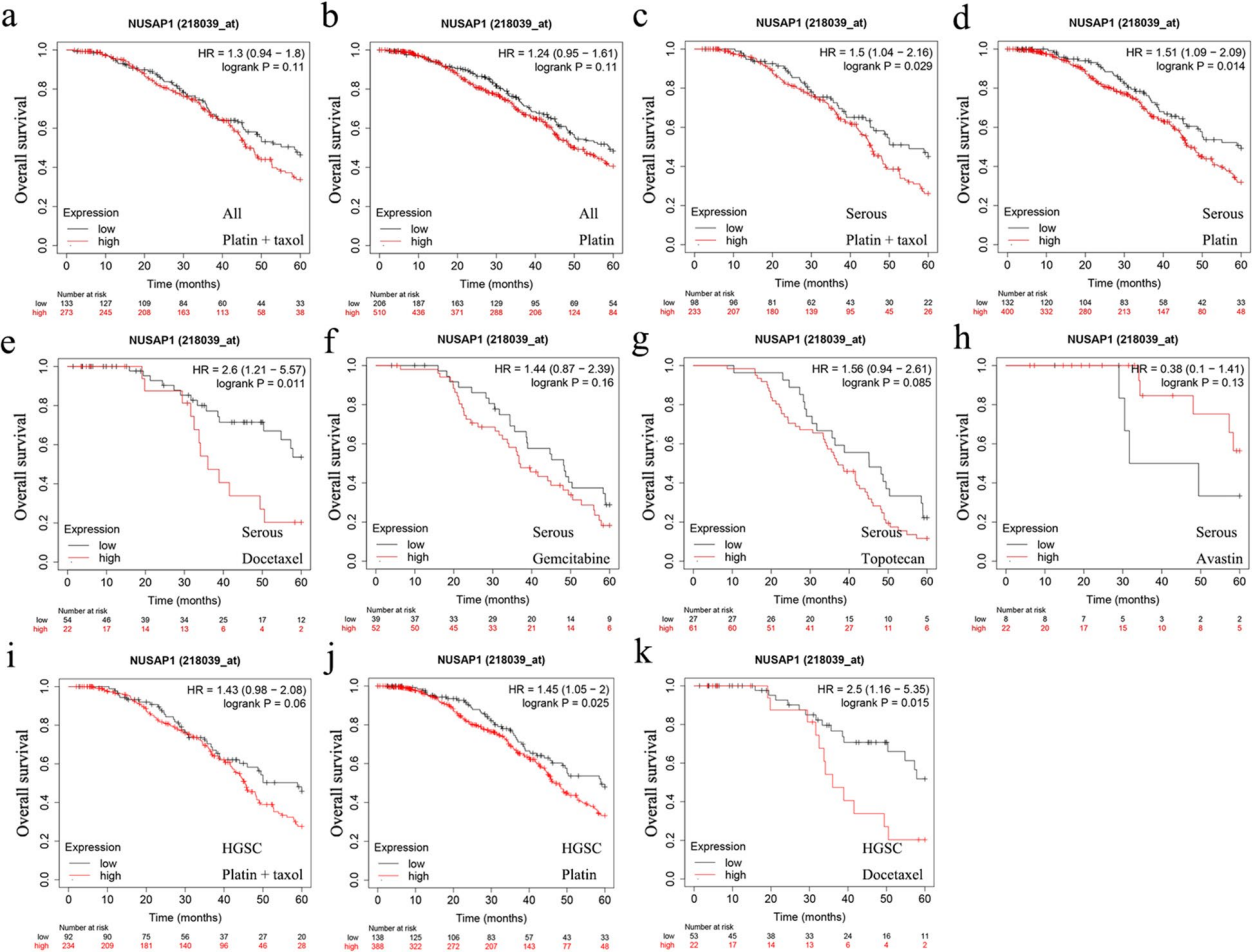
Relationship between *NUSAP1* expression and chemotherapy in patients with ovarian cancer. **a–b** Prognostic significance of *NUSAP1* in patients with ovarian cancer treated with platinum-paclitaxel combination or platinum chemotherapy. **c–h** Prognostic significance of *NUSAP1* in patients with serous ovarian cancer treated with platinum-paclitaxel combination or other monotherapies. **i-k** Prognostic significance of *NUSAP1* in patients with high-grade serous ovarian cancer treated with platinum-paclitaxel combination or other monotherapies

We further analyzed the correlation between the expression of *NUSAP1* and the prognosis of chemotherapy after optimal surgery. For all patients with ovarian cancer, *NUSAP1* could not predict the prognosis of receiving platinum-paclitaxel combination or platinum chemotherapy (Fig. 3a, b). Surprisingly, serous ovarian cancer patients with high *NUSAP1* expression had a poor prognosis after receiving these chemotherapy regimens (Fig. 3c, d). Similar results were obtained when patients were treated with docetaxel (Fig. 3e). However, *NUSAP1* had no effect on the prognosis of patients with serous ovarian cancer treated with gemcitabine, topotecan, or avastin (Fig. 3f, g, h). As for patients with HGSC, upregulation of *NUSAP1* was correlated with poor prognosis after platinum or docetaxel therapy (Fig. 3i, j, k). Therefore, *NUSAP1* upregulation may be related to platinum and docetaxel-based chemotherapy resistance in patients with serous ovarian cancer.

### NUSAP1 is highly expressed in ovarian epithelial malignancies

**Fig. 4:**
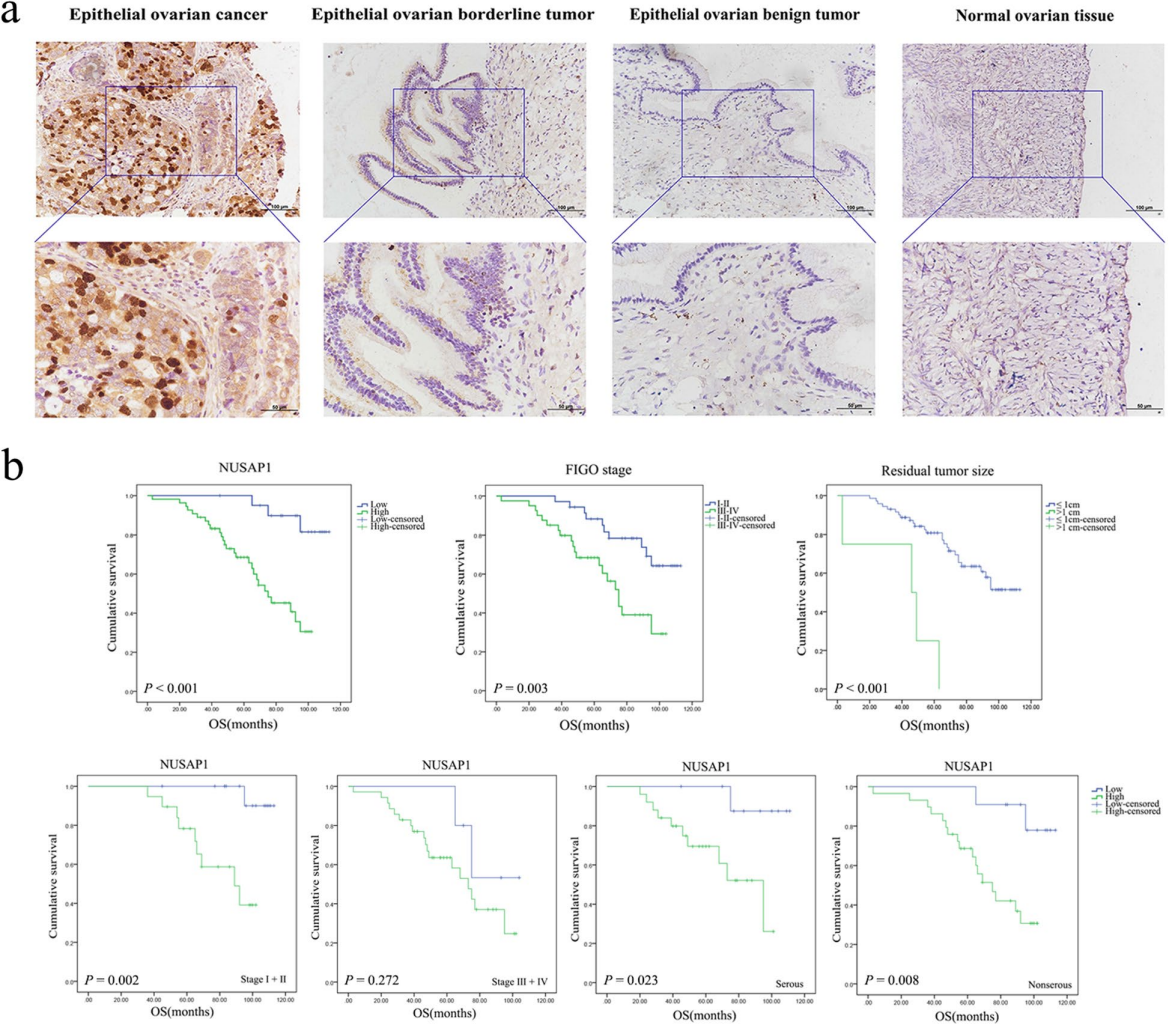
NUSAP1 expression and prognosis value in ovarian epithelial malignancies. **a** Representative images of NUSAP1 expression in ovarian tissue: epithelial ovarian cancer, epithelial ovarian borderline tumor, benign ovarian epithelial tumor, and normal ovarian tissue. Scale bar: 100 μm (upper picture) and 50 μm (lower picture). The lower picture is an enlarged view of the blue box in the upper picture. **b** Correlation between NUSAP1 expression, FIGO stage, residual tumor size, and survival prognosis in patients with epithelial ovarian malignancies

NUSAP1 expression in our tissues cohort was evaluated by IHC assay. Results showed that NUSAP1 was mainly expressed in the cytoplasm and nucleus. The positive rate and high-positive rate of the malignant tumor group were significantly higher than those of the borderline, benign, and normal group. No significant differences in NUSAP1 expression were observed among borderline, benign and normal tissues (Fig. 4a; Table 1).

**Table 1:** Statistical analysis of NUSAP1 expression in ovarian tissues from different groups

**Group**	***n***	**Low**		**High**		**Positive rate (%)**	**High positive rate (%)**
		**(-)**	**( +)**	**(+ +)**	**(+ + +)**		
Normal	12	10	1	1	0	16.67	8.33
Benign	10	6	3	0	1	40.00	10.00
Borderline	14	6	4	2	2	57.14	28.57
Malignant	78	7	15	27	29	91.03*	71.79*

^*^ *P* < 0.05

### NUSAP1 expression in epithelial ovarian malignant tumors is related to the FIGO stage

Then, we evaluated the association between NUSAP1 expression and clinicopathological characteristics (Table 2). Seventy-eight patients with ovarian epithelial malignant tumors were divided into two groups according to the NUSAP1 expression level. High NUSAP1 expression had a notable correlation with FIGO stage. The high-positive rate of NUSAP1 expression was significantly higher in the patients with FIGO III-IV compared with that in the patients with FIGO I-II. NUSAP1 showed the highest positive rate in ovarian clear cell carcinoma. In addition, the high-positive rate of NUSAP1 in HGSC was higher than that in low-grade serous ovarian cancer. However, NUSAP1 level was not significantly correlated with tumor grade, lymph node metastasis, residual tumor size, or pathological type.

**Table 2:** Relationship between NUSAP1 expression level in epithelial ovarian cancer and clinicopathological parameters

Characteristics	*n*	Low		High		High positive rate (%)	*P*-value
		**(-)**	**( +)**	**(+ +)**	**(+ + +)**		
**FIGO stage**							
I-II	34	5	10	9	10	55.88	0.006*
III-IV	44	2	5	18	19	84.09	
**Tumor grade**							
1–2	43	4	12	13	14	62.79	0.050
3	35	3	3	14	15	82.86	
**LN metastasis**							
No	33	4	7	9	13	66.67	> 0.050
Yes	18	0	3	8	7	83.33	
No lymphadenectomy	27	3	5	10	9	70.37	
**Residual tumor size**							
≤ 1 cm	74	7	14	25	28	71.62	> 0.050
> 1 cm	4	0	1	2	1	75.00	
**Pathologic type**							
High-grade serous	43	4	5	14	20	79.07	> 0.050
Low-grade serous	9	1	3	2	3	55.56	
Mucinous	8	2	2	2	2	50.00	
Endometrioid	12	0	5	4	3	58.33	
Clear cell	6	0	0	5	1	100.00	

^*^ *P* < 0.05

### High NUSAP1 expression is an independent risk factor for the prognosis of patients with ovarian epithelial malignant tumors

We followed-up 78 patients with ovarian epithelial malignant tumors (last follow-up time was September 30, 2017), 65 of whom (83.33%) had complete clinical information, whereas 13 cases (16.67%) were lost to follow-up. Kaplan**–**Meier and log-rank tests showed that the 5-year survival rates of patients with high NUSAP1 expression, FIGO III-IV, and residual tumor size > 1 cm were significantly decreased (Fig. 4b). In the multivariate Cox′s proportional hazards model analysis, NUSAP1 expression level, FIGO stage, and residual tumor size were found to be independent prognostic factors (Table 3).

**Table 3:** Cox regression model analysis of overall survival in patients with epithelial ovarian cancer

Variables	Univariate analysis			Multivariate analysis		
	**HR**	**95% CI of HR**	***P-*value**	**HR**	**95% CI of HR**	***P-*value**
NUSAP1 expression (low vs. high)	6.802	2.039–22.693	0.002*	4.463	1.268–15.704	0.020*
Age (years) (< 60 vs. ≥ 60)	1.886	0.914–3.891	0.086	2.067	0.965–4.427	0.062
FIGO stage (I-II vs. III-IV)	3.043	1.404–6.596	0.005*	2.572	1.005–6.579	0.049*
Tumor grade (1–2 vs. 3)	1.405	0.684–2.886	0.354	0.925	0.414–2.065	0.849
Lymph node metastasis (no vs. yes)	1.702	0.754–3.846	0.201	1.159	0.488–2.753	0.737
Residual tumor size (≤ 1 cm vs. > 1 cm)	7.739	2.503–23.929	0.000*	4.697	1.453–15.188	0.010*
Pathological subtype (nonserous vs. serous)	1.208	0.574–2.546	0.619	2.087	0.848–5.140	0.110

^*^P < 0.05

To investigate the significance of NUSAP1 in epithelial ovarian cancer of different stages, we analyzed the prognostic significance stratified by FIGO stage. Both Kaplan–Meier survival analysis and Cox regression analysis showed that high expression of NUSAP1 was associated with shorter OS in early-stage, but not in advanced-stage epithelial ovarian cancer (Fig. 4b, Table 4). Therefore, the prognostic significance of NUSAP1 mainly exists in early-stage.

**Table 4:** Cox regression model analysis of overall survival in patients with early-stage and advanced-stage epithelial ovarian cancer

Variables	Univariate analysis			Multivariate analysis		
	**HR**	**95% CI of HR**	***P-*value**	**HR**	**95% CI of HR**	***P-*value**
**Early-stage epithelial ovarian cancer**						
NUSAP1 expression (low vs. high)	12.697	1.587–101.576	0.017*	9.522	1.064–85.252	0.044*
Age (years) (< 60 vs. ≥ 60)	2.046	0.575–7.290	0.269	1.451	0.395–5.337	0.575
Tumor grade (1–2 vs. 3)	1.708	0.493–5.914	0.398	0.859	0.216–3.411	0.829
Lymph node metastasis (no vs. yes)	-	-	-	-	-	-
Residual tumor size (≤ 1 cm vs. > 1 cm)	-	-	-	-	-	-
Pathological subtype (nonserous vs. serous)	29.281	0.044–19,460.028	0.308	111,520.722	0.000–3.960E + 258	0.969
**Advanced-stage epithelial ovarian cancer**						
NUSAP1 expression (low vs. high)	2.223	0.511–9.664	0.287	2.234	0.471–10.585	0.311
Age (years) (< 60 vs. ≥ 60)	1.621	0.670–3.920	0.284	2.262	0.876–5.839	0.092
Tumor grade (1–2 vs. 3)	1.022	0.415–2.517	0.963	0.821	0.282–2.394	0.718
Lymph node metastasis (no vs. yes)	1.013	0.402–2.552	0.979	1.093	0.417–2.867	0.857
Residual tumor size (≤ 1 cm vs. > 1 cm)	4.894	1.503–15.940	0.008*	4.604	1.371–15.466	0.014*
Pathological subtype (nonserous vs. serous)	1.747	0.723–4.223	0.215	1.982	0.698–5.629	0.199

^*^P < 0.05

Ovarian serous cancer is the most common pathological type of ovarian cancer. Therefore, we focused on the prognostic value of NUSAP1 in the specific pathological subtype. Kaplan–Meier survival analysis showed that for patients with serous and non-serous ovarian cancer, high expression of NUSAP1 was associated with poor OS (Fig. 4b). By univariate analysis, NUSAP1 expression and residual tumor size predicted poor OS in serous ovarian cancer. However, for patients with HGSC, the Cox regression model only suggested the correlation between residual tumor size and prognosis (Table 5).

**Table 5:** Cox regression model analysis of overall survival in patients with serous ovarian cancer

Variables	Univariate analysis			Multivariate analysis		
	**HR**	**95% CI of HR**	***P-*value**	**HR**	**95% CI of HR**	***P-*value**
**Serous ovarian cancer**						
NUSAP1 expression (low vs. high)	8.234	1.027–65.981	0.047*	6.339	0.644–62.387	0.113
Age (years) (< 60 vs. ≥ 60)	3.381	0.969–11.802	0.056	2.564	0.657–10.005	0.175
FIGO stage (I-II vs. III-IV)	39.941	0.189–8433.010	0.177	233,779.033	0.000–1.579E + 217	0.960
Tumor grade (1–2 vs. 3)	0.398	0.085–1.854	0.240	0.370	0.067–2.044	0.254
Lymph node metastasis (no vs. yes)	1.370	0.397–4.726	0.619	1.093	0.417–2.867	0.857
Residual tumor size (≤ 1 cm vs. > 1 cm)	5.800	1.119–30.054	0.036*	1.845	0.428 -7.948	0.411
**HGSC**						
NUSAP1 expression (low vs. high)	5.851	0.706–48.468	0.101	1.648	0.161–16.852	0.674
Age (years) (< 60 vs. ≥ 60)	3.463	0.816–14.692	0.092	1.969	0.391–9.907	0.411
FIGO stage (I-II vs. III-IV)	36.733	0.057–23,576.497	0.266	200,211.029	0.000–1.573E + 287	0.971
Lymph node metastasis (no vs. yes)	0.357	0.044–2.913	0.336	0.296	0.026–3.353	0.325
Residual tumor size (≤ 1 cm vs. > 1 cm)	13.416	1.861–96.709	0.010*	12.161	1.329–111.235	0.027*

Abbreviation: *HGSC* High-grade serous ovarian cancer. Note. *P < 0.05

### Molecular mechanism of NUSAP1 involved in ovarian cancer development

**Fig. 5:**
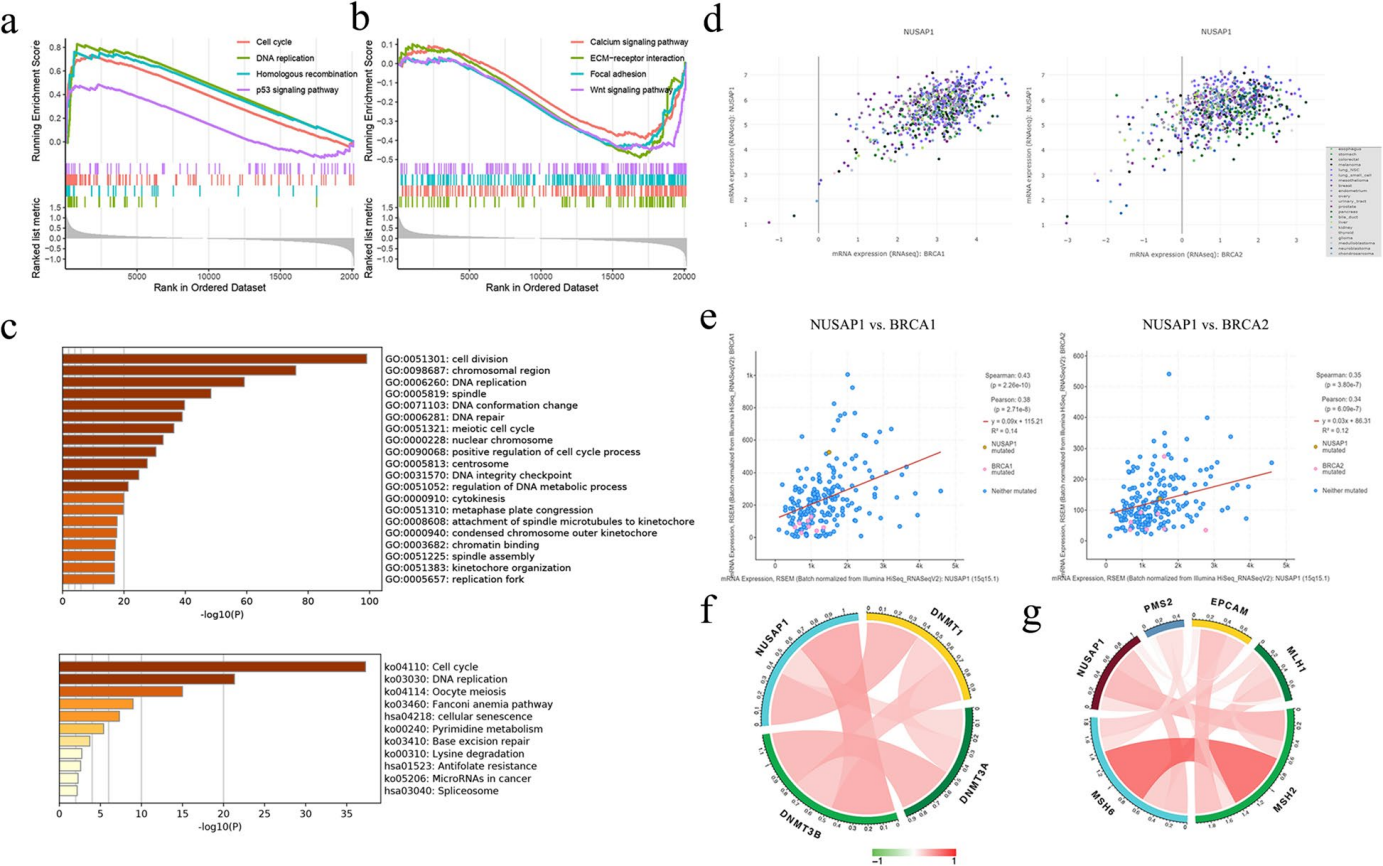
Molecular mechanism of *NUSAP1* in the development of ovarian cancer. **a** Gene set enrichment analysis of *NUSAP1* high-expressing samples. **b** Gene set enrichment analysis of *NUSAP1* low-expressing samples. **c** Functional and pathway enrichment analysis of *NUSAP1* based on Metascape data. **d** Correlation between *NUSAP1* and *BRCA1/2* expression in multiple cancer cell lines according to the CCLE database. **e** Correlation between *NUSAP1* and *BRCA1/2* expression in ovarian cancer according to the cBioPortal database. **f** Correlation between *NUSAP1* and methyltransferases. **g** Correlation between *NUSAP1* and MMR key genes. Color depth is positively related to correlation

To explore the relevant pathways in which *NUSAP1* is involved, we conducted gene set enrichment analysis. The results indicated that the cell cycle, DNA replication, homologous recombination, and p53 signaling pathway were enriched in *NUSAP1* high-expressing samples (Fig. 5a). Calcium signaling pathway, extracellular matrix-receptor interaction, focal adhesion, and the Wnt signaling pathway were enriched in *NUSAP1* low-expressing samples (Fig. 5b). The Metascape database also confirmed that *NUSAP1* and its co-expressed genes were involved in the regulation of DNA repair (GO: 0006281), replication fork (GO: 0005657), and base excision repair (ko03410) (Fig. 5c).

Homologous recombination repair (HRR) is a key repair mechanism for DNA double-strand breaks, in which *BRCA1/2* participates as key tumor suppressor genes. The correlation between *NUSAP1* and *BRCA1/2* was analyzed through the CCLE and cBioPortal databases. Both databases showed a positive correlation between *NUSAP1* and *BRCA1/2* expression (Fig. 5d, e). In addition, studies confirmed that DNA methyltransferase (DNMT) inhibitors induced a BRCAness phenotype. We further analyzed the correlation between *NUSAP1* and methyltransferase, and found that *NUSAP1* was positively correlated with *DNMT1*, *DNMT3A*, and *DNMT3B* (Fig. 5f).

The mismatch repair (MMR) function is responsible for repairing DNA base mismatches that occur during replication. Mutation of MMR-related genes will cause DNA replication errors, thereby resulting in genetic instability and higher somatic mutations. We analyzed the correlation between *NUSAP1* and key MMR genes, among them, *NUSAP1* was positively correlated with *MSH2* and *MSH6* (Fig. 5g).

### Correlation between NUSAP1 expression and immune cells in ovarian cancer

**Fig. 6:**
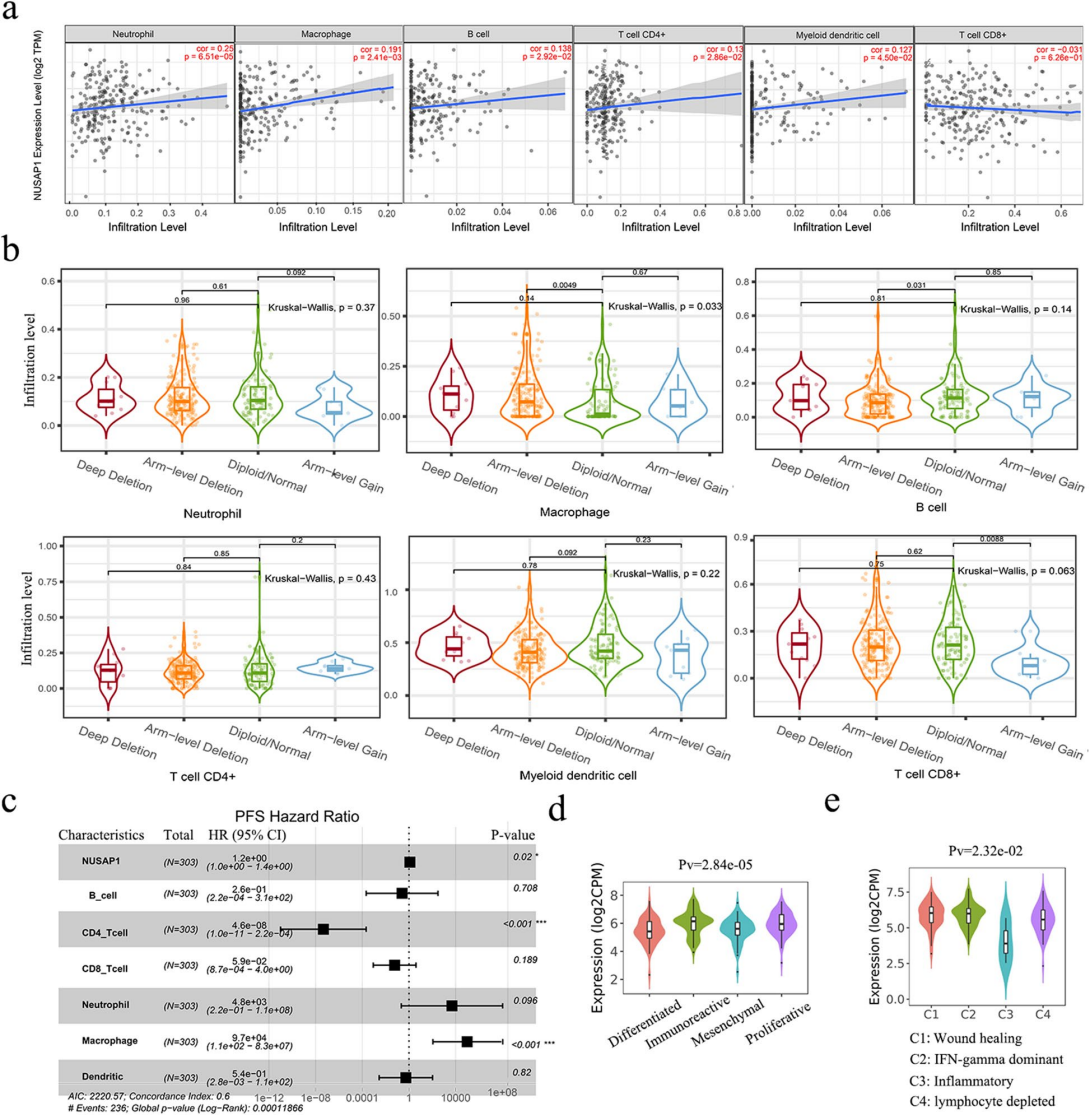
Relationship between *NUSAP1* and immune cell infiltration in ovarian cancer. **a** Correlation between *NUSAP1* expression and immune cell infiltration. **b** Comparison of immune cell infiltration in ovarian cancers with different *NUSAP1* somatic copy number alterations. The shape of the violin plot reflects the distribution of various immune cell infiltrations in the different groups. The number in the plot reflects the *P*-value between two groups. **c** Forest plot of the multivariate Cox regression analysis of PFS in patients with ovarian cancer. **d** Distribution of *NUSAP1* expression across ovarian cancer molecular subtypes derived from TCGA datasets. **e** Distribution of *NUSAP1* expression across ovarian cancer immune subtypes derived from TCGA datasets

In order to explore the predictive value of *NUSAP1* in ovarian cancer immunotherapy response, we analyzed the relationship between *NUSAP1* and immune infiltration. The Timer database showed that high expression of *NUSAP1* indicated high infiltrating abundances of neutrophils, macrophages, B cells, CD4 + T cells, and myeloid dendritic cells (Fig. 6a). We further compared the tumor infiltration level in ovarian cancer with different somatic copy number variations of *NUSAP1* (Fig. 6b). Compared with arm-level gain, normal copy number of *NUSAP1* was correlated with increased infiltration of CD8 + T cells, although the correlation between *NUSAP1* expression and CD8 + T cells infiltration level was not significant.

Furthermore, multivariable hazards models were used to evaluate the impact of *NUSAP1* expression in the presence of varying immune cells. *NUSAP1* had 1.2-fold higher risk on PFS (Fig. 6c).

Moreover, we explored the distribution of *NUSAP1* expression across molecular subtypes in ovarian cancer using the TISIDB database. TCGA ovarian samples were classified into differentiated, immunoreactive, mesenchymal, and proliferative subtypes. The results showed that the expression of *NUSAP1* was relatively high in immunoreactive and proliferative subtypes (Fig. 6d). Further analysis of immune subtypes showed that the expression of *NUSAP1* was the lowest in the inflammatory (C3) subtype (Fig. 6e). Hence, NUSAP1 could be a potential biomarker for immunotherapy response prediction.

## Discussion

Ovarian cancer currently lacks effective early screening and diagnostic methods and is prone to recurrence, metastasis, and drug resistance, resulting in no significant improvement in the 5-year OS of affected patients. Therefore, finding valuable tumor markers and exploring their contribution for ovarian cancer development are crucial for the diagnosis, treatment, and prognosis of patients with ovarian cancer. In this study, the expression of NUSAP1 was validated though multiple databases. IHC assay confirmed that NUSAP1 is highly expressed in ovarian cancer and is an independent risk factor for patient survival prognosis. Furthermore, database analyses indicated molecular mechanisms through which NUSAP1 may be involved in ovarian cancer development.

Numerous studies have confirmed that NUSAP1 is upregulated in breast cancer [8], hepatocellular carcinoma [9], and other malignant tumors, and is closely related to malignant biological behaviors, such as cell cycle regulation, invasion, and metastasis. NUSAP1 silencing inhibited DNMT1 expression, leading to the inhibition of liver and colorectal cancer progression [9]. NUSAP1 promotes the nuclear translocation of GLI family zinc finger 1 and activates the Hedgehog signaling pathway, resulting in increased aggressiveness of astrocytoma [12]. Li et al. found that NUSAP1 promotes metastasis and induces cancer stem cell-like properties of cervical cancer cells by activating Wnt/β-catenin signals [15]. Moreover, Xu et al. demonstrated that inhibition of NUSAP1 suppresses cell growth and metastasis by regulating the BTG2/PI3K/Akt signals in non-small-cell lung cancer [29]. These studies indicate that NUSAP1 is closely related to the occurrence of various tumors, and its high expression may promote the malignant biological behavior of tumors. Recently, Shen et al. proposed six hub genes, including *NUSAP1*, to predict the development and prognosis of ovarian cancer based on the Gene Expression Omnibus data. However, this study did not conduct experiments to verify the results of the bioinformatics analysis and evaluate the contribution of each hub gene separately [30]. Zhang et al. showed that knocking down NUSAP1 in ovarian cancer cells promotes apoptosis and affects the cell cycle distribution [31]. However, its specific mechanism of function in ovarian cancer has not been clarified. Herein, through the joint analysis of Oncomine, TCGA, and CCLE databases, we found that *NUSAP1* is highly expressed in multiple malignant tumors, and its protein level is correlated with FIGO stage. Results of IHC assay showed that the positive rate of NUSAP1 in ovarian malignant tumors is significantly higher than that in other groups. In addition, a significant correlation between NUSAP1 level and FIGO stage was observed. Further analysis of the expression of NUSAP1 in different histological types of ovarian cancer showed that NUSAP1 had the highest positive rate in ovarian clear cell carcinoma. However, due to the limitation of the number of clear cell carcinoma cases, it is necessary to expand the sample size to further verify the conclusion.

High NUSAP1 expression predicts poor prognosis in astrocytoma [12], breast cancer [13], esophageal squamous cell carcinoma [10], and colon cancer [14]. However, opposite results were obtained in the prognostic analysis of cervical cancer [15, 32]. In addition, studies have suggested a correlation between NUSAP1 and chemotherapeutic resistance. In oral squamous cell carcinoma, NUSAP1 downregulation enhances the anti-tumor effect of paclitaxel by activating apoptotic pathways [33]. Moreover, Zhang et al. reported that knockdown of NUSAP1 increased susceptibility to epirubicin in invasive breast cancer [34]. Recent investigations showed that NUSAP1 stabilizes the ATR by sumoylation via its SAP domain, thereby promoting chemotherapeutic resistance to temozolomide and doxorubicin [35]. In this study, we followed up cancer patients for more than 5 years to explore the prognostic value of NUSAP1. The result of Cox regression analysis, which was consistent with the results of bioinformatics analysis, showed that high NUSAP1 expression conferred poor prognosis of patients with ovarian cancer. In addition, NUSAP1 showed an even more significant correlation with prognosis in patients with early-stage. Ovarian serous cancer is the most common pathological type of ovarian cancer, and HGSC is a leading cause of ovarian cancer-related death [36]. Five years after diagnosis, advanced-stage HGSC cases had a survival rate of 32.1% [37]. The results of bioinformatics analysis and immunohistochemistry statistics indicated that high expression of NUSAP1 was correlated with poor OS of patients with serous ovarian cancer. However, for the patients with HGSC, our clinical sample analysis results were not consistent with the database, which may be limited by the number of cases and the heterogeneity of the study cohort.

We also explored the molecular mechanism of *NUSAP1* contributing for the tumorigenesis and progression of ovarian cancer. Database analysis showed that *NUSAP1* is involved in the regulation of the DNA repair, homologous recombination, base excision repair, replication fork, cell cycle, DNA replication, and p53 signaling pathway. Studies have confirmed that these pathways are closely related to ovarian cancer. NUSAP1 has been confirmed to help generate microtubules near chromatin, leading to chromatin-induced spindle formation, which is essential for cell proliferation [38]. In particular, Mills et al. identified NUSAP1 as a substrate for SCF-type E3 ubiquitin ligase during S/G2 phase of the cell cycle and observed a cell cycle regulatory interaction between NUSAP1 and a SUMO (small ubiquitin-like modifier) E3 ligase complex through mass spectrometry-based proteomics [16, 39]. In addition, inhibition of NUSAP1 expression reduced cyclin A2 and cyclin B1 levels, and induced cell cycle arrest [10, 15, 40].

Several types of DNA damage repair (DDR) pathways, including nucleotide excision repair, base excision repair, MMR, homologous recombination, and non-homologous end joining, can detect DNA damage and initiate the process of cell repair. Hence, alterations in DDR pathways lead to genomic instability and cancer development [41]. As an error-free repair mechanism, HRR is a key repair method for DNA double-strand breaks. Epigenetic modifications and mutations in HRR-related genes can cause homologous recombination deficiency, thereby enhancing the sensitivity of patients to PARP inhibitors [42]. Studies showed that NUSAP1 knockdown reduces the sumoylation level of ATR and cause DNA damage in glioblastoma multiforme cells [35]. In addition, NUSAP1, similar to BRCA1, plays a role in DNA double-stand break repair via the homologous recombination and single-strand annealing pathways in breast cancer, and it protects BRCA1 from proteasome-mediated degradation [8]. The combination of NUSAP1 and BRCA1 improves the prognostic power in triple-negative breast cancer compared with the calculation based on age, menstrual status, and lymph node status [13]. We found that *NUSAP1* is correlated with DNA repair, replication fork and homologous recombination in ovarian cancer. In addition, both the CCLE and cBioPortal database showed a positive correlation between the expression of *NUSAP1* and *BRCA1* in ovarian cancer. Therefore, NUSAP1 in ovarian cancer may participate in HRR by regulating the levels of ATR and BRCA1/2; thus, low expression of NUSAP1 in ovarian cancer implies sensitivity to PARP inhibitors. Studies have shown that the acquisition of a drug resistant phenotype is related to the accumulation of epigenetic variations in tumor suppressor and DNA repair genes [41]. DNMTs are a class of epigenetic regulatory enzymes, of which DNMT1 is involved in maintaining the methylation status after DNA synthesis and DNMT3A/B are involved in the de novo methylation of DNA [43]. It was reported that DNMT inhibitors induce a BRCAness phenotype through the downregulation of homologous recombination and NHEJ genes, thereby sensitizing non-small cell lung cancer cells to PARP inhibitors [44]. In addition, combination of DNMT inhibitors and PARP inhibitors promotes the accumulation of reactive oxygen species (ROS), thereby sensitizing breast and ovarian cancer cells to PARP inhibitors in a ROS-cAMP/PKA-dependent manner [45]. Therefore, we further evaluated the correlation between *NUSAP1* and methyltransferases, and found that *NUSAP1* was positively correlated with *DNMT1*, *DNMT3A* and *DNMT3B*. Therefore, patients with high expression of NUSAP1 can be treated with the combination of DNMT inhibitors and PARP inhibitors to enhance treatment efficacy and overall survival.

The MMR system is responsible for repairing DNA base mismatches [46]; thus, MMR gene mutations and hypermethylation in the promoter region will result in microsatellite instability (MSI), which are mostly caused by inactivation of *MLH1*, *PMS2*, *MSH2*, or *MSH6* [47]. Solid tumors with deficient MMR usually have immunogenicity and extensive T-cell infiltration, which make them more responsive to the treatment with immune checkpoint inhibitors (ICIs) [48, 49]. Although the response rate to ICIs in ovarian cancer is usually lower than in other cancer types, a proportion of ovarian cancers exhibiting MSI show an increased number of CD3 + and CD8 + tumor infiltrating lymphocytes [50, 51]. Herein, we analyzed the correlation between *NUSAP1* and key MMR genes, which revealed that *NUSAP1* is correlated with *MSH2* and *MSH6*. Furthermore, we analyzed the correlation between *NUSAP1* and immune infiltration level. *NUSAP1* showed a strongest correlation with neutrophil tumor infiltration. Neutrophils are the most abundant white blood cells and are closely related to inflammation; however, the role and characteristics of neutrophils in cancer are controversial. Studies showed that neutrophils have more anti-tumor phenotype during early stages of tumor progression, whereas become more tumorigenic during aggressive stages [52, 53]. Neutrophils maintain tumor growth by suppressing T cell activation, accelerating gene mutation, tumor cell proliferation, angiogenesis and metastasis [54]. It has been observed that high infiltration with tumor-associated neutrophils is associated with poor prognosis in multiple cancers [55]. Comprehensive meta-analysis showed that high neutrophil-to-lymphocyte ratio is associated with shorter PFS and OS in ovarian cancer [56]. In addition, the infiltration of CD8 + T cells in tumors was found to be associated with favorable prognosis in ovarian cancer [57]. Therefore, high *NUSAP1* expression may cause increased infiltration of neutrophils and decreased infiltration of CD8 + T cells, leading to the progression and poor prognosis of ovarian cancer. Altogether, NUSAP1 may represent a new biomarker for predicting the efficacy of immunotherapy in ovarian cancer.

## Conclusions

In summary, database analyses and IHC assay confirmed that NUSAP1 is highly expressed in ovarian cancer. High NUSAP1 level is an independent risk factor affecting the survival and prognosis of patients with ovarian cancer, especially for patients with early-stage or serous ovarian cancer. Moreover, NUSAP1 expression is closely related to response to chemotherapy. Through multi-omics analysis, we confirmed that high *NUSAP1* expression is closely related to signaling pathways that influence the development of cancer, such as cell cycle, DNA replication, homologous recombination, and p53 signals. In addition, a correlation was found between the expression of *NUSAP*1 and immune cell infiltration. Therefore, this study highlights the significant value of NUSAP1 for predicting prognosis and response to chemotherapy and immunotherapy in ovarian cancer. Further investigation using in vitro and in vivo models are needed to validate the biological significance of NUSAP1 in different pathological subtypes of ovarian cancer and to elucidate the molecular mechanism of NUSAP1 in regulating DNA repair. In addition, the ability of NUSAP1 as a biomarker to predict the efficacy of PARP inhibitors alone or in combination with DNMT inhibitions warrants further evaluation, which will assist in the development of future individualized therapeutic strategies for ovarian cancer.

## Data Availability

The datasets generated by the TCGA Research Network during the current study are available on the website, https://www.cancer.gov/tcga. Other data generated or analysed during this study are included in this published article.

## Abbreviations

CCLE: Cancer Cell Line Encyclopedia
CI: Confidence interval
CPTAC: Clinical Proteomic Tumor Analysis Consortium
DDR: DNA damage repair
DNMT: DNA methyltransferase
FIGO: International Federation of Gynecology and Obstetrics
GSEA: Gene set enrichment analysis
HGSC: High-grade serous ovarian cancer
HPA: Human Protein Atlas
HR: Hazard ratio
HRR: Homologous recombination repair
ICI: Immune checkpoint inhibitor
IHC: Immunohistochemistry
MMR: DNA mismatch repair
MSI: Microsatellite instability
NHEJ: Non-homologous end joining
NUSAP1: Nucleolar and spindle-associated protein 1
OS: Overall survival
PBS: Phosphate-buffered saline
PFS: Progression-free survival
ROS: Reactive oxygen species
SUMO: Small ubiquitin-like modifier
TCGA: The Cancer Genome Atlas
